# Adipokines and Their Role in Heart Failure: A Literature Review

**DOI:** 10.19102/icrm.2023.14111

**Published:** 2023-11-15

**Authors:** Saira Rafaqat

**Affiliations:** 1 Department of Zoology (Molecular Physiology), Lahore College for Women University, Lahore, Pakistan

**Keywords:** Adipokines, heart failure, pathogenesis

## Abstract

Obesity is a major risk factor for heart failure (HF). The relationship between adipokines and HF has been implicated in many previous studies and reviews. However, this review article summarizes the basic role of major adipokines, such as apelin, adiponectin, chemerin, resistin, retinol-binding protein 4 (RBP4), vaspin, visfatin, plasminogen activator inhibitor-1, monocyte chemotactic protein-1, nesfatin-1, progranulin, leptin, omentin-1, lipocalin-2, and follistatin-like 1 (FSTL1), in the pathogenesis of HF. Apelin is reduced in patients with HF and upregulated following favorable left ventricular (LV) remodeling. Higher levels of adiponectin have been found in patients with HF compared to in control patients. Also, high plasma chemerin levels are linked to a higher risk of HF. Serum resistin is related to the severity of HF and associated with a high risk for adverse cardiac events. Evidence indicates that RBP4 can contribute to inflammation and damage heart muscle cells, potentially leading to HF. Vaspin might stop the progression of cardiac degeneration, fibrosis, and HF according to experiments on rats with experimental isoproterenol-induced chronic HF. The serum concentrations of visfatin are significantly lower in patients with systolic HF. Leptin levels were found to be correlated with low LV mass and myocardial stiffness, both of which are significant risk factors for the development of HF with preserved ejection fraction (HFpEF). Measuring serum omentin-1 levels appears to be a novel prognostic indicator for risk stratification in HF patients. Increased expression of neutrophil gelatinase–associated lipocalin in both systemic circulation and myocardium in clinical and experimental HF suggests that innate immune responses may contribute to the development of HF. FSTL1 was elevated in patients with HF with reduced ejection fraction and associated with an increase in the size of the left ventricle of the heart. However, other adipokines, such as plasminogen activator inhibitor-1, monocyte chemotactic protein-1, nesfatin-1, and progranulin, have not yet been studied for HF.

## Introduction

Heart failure (HF) is a major contributor to morbidity, hospitalization, and mortality among elderly individuals. It is a growing public health issue that places a substantial financial burden on the health care system.^1^ For individuals >65 years of age, chronic heart failure (CHF) is the primary reason for hospitalization and has a significant clinical and economic impact. Roughly 50% of hospital readmissions are associated with CHF-related comorbidities, polypharmacy, and disabilities.^2^

There is a growing proportion of individuals, particularly older women who have diabetes, obesity, and atrial fibrillation, who are experiencing HF with preserved systolic function. Various mechanisms contribute to HF, including increased stress on the heart, dysfunction related to a lack of blood flow, changes in the structure of the heart, excessive stimulation of the nervous and hormonal systems, abnormalities in the way the heart cells use calcium, excessive or insufficient growth of the extracellular matrix, accelerated cell death, and genetic mutations. Biomarkers that are released due to the stretching of the heart muscle, imbalances between the formation and breakdown of the extracellular matrix, inflammation, and kidney failure can help identify the cause of HF. When considered together, these biomarkers may assist in predicting the prognosis and selecting the appropriate treatment.^3^

Despite significant advancements in the treatment of many cardiac conditions, HF continues to be a significant health concern, with its incidence and prevalence on the rise over the past few decades. As a result, there has been a considerable increase in the number of potential biomarkers that could aid in the diagnosis and treatment of patients with HF. Currently, several biomarkers are ready for everyday clinical use, and they reflect the various underlying pathophysiological processes present in HF. The identification of various biomarkers linked to the diagnosis and prognosis of HF in recent years has improved the treatment and risk stratification of HF patients.^4^

Obesity is presently recognized as a persistent inflammatory state that operates at a low level. Inflammation-triggering molecules called pro-inflammatory cytokines are increased in people with obesity, including those generated by adipose tissue, either by the fat cells themselves or the macrophages that penetrate the fat tissue, and these molecules are referred to as adipokines.^5^ Over the last 30 years, there has been a significant increase in obesity rates, posing a threat to the health of people in both developed and developing countries. Obesity, characterized by the accumulation of visceral fat, is a major factor in the clustering of metabolic disorders like type 2 diabetes, dyslipidemia, and hypertension, ultimately leading to the development of cardiovascular disease (CVD). Adipose tissue, in addition to being an energy storage organ, is also an active endocrine tissue that produces several biologically active proteins called adipokines.^6^

The occurrence of CVD and the emergence of CHF are both linked to obesity as recognized risk factors.^7^ Obesity is becoming a prevalent issue across the globe. Medical experts are now more aware of the myocardial changes that occur in individuals who are obese, regardless of whether they have hypertension, obstructive sleep apnea (OSA), or coronary artery disease. Studies show that being obese in young adulthood significantly increases the risk of developing both ischemic heart disease (IHD) and congestive heart failure, even without pre-existing IHD.^8,9^ In patients with HF, grip strength and bone mineral density were linked to adipokines. The disruption of adipokine balance may contribute to the onset of frailty in HF.^10^

## Methods

The relationship between adipokines and HF has been implicated in many previous studies and reviews. This review article focused on a few major adipokines that are not well described in HF. However, we summarize the potential role of major adipokines, such as apelin, adiponectin, chemerin, resistin, retinol-binding protein 4 (RBP4), vaspin, visfatin, plasminogen activator inhibitor-1, monocyte chemotactic protein-1, nesfatin-1, progranulin, leptin, omentin-1, lipocalin-2 (LCN-2), and follistatin-like 1 (FSTL1), in the pathogenesis of HF.

Various databases, including Google Scholar, PubMed, and ScienceDirect, were used to examine the literature. The literature search was conducted until December 1, 2022. Keywords such as “adipokines,” “heart failure,” “apelin,” “adiponectin,” “chemerin,” “resistin,” “retinol-binding protein 4,” “vaspin,” “visfatin,” “leptin,” “omentin-1,” “lipocalin-2,” and “follistatin-like 1” were used. Only clinical studies written in English were considered. Although more recent studies were given preference, no specific limit on publication date was set. Furthermore, the references in the pertinent articles were evaluated, and related articles were also identified.

## An overview of obesity in the pathogenesis of heart failure

**Figure 1:**
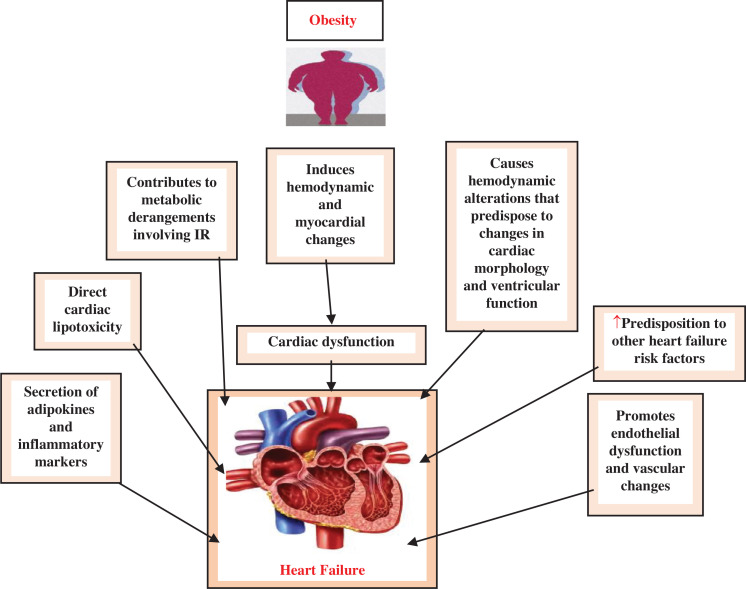
Some major aspects of obesity involved in the pathogenesis of HF. ↑ denotes increased levels. *Abbreviations:* HF, heart failure; IR, insulin resistance.

**Figure 1** explains the few major aspects of obesity involved in the pathogenesis of HF. Apart from the effects on adipokine signaling, obesity can also contribute to HF through other mechanisms like neurohormonal activation, heightened oxidative stress,^11,12^ accumulation of free fatty acids in myocytes,^13^ and depletion of B-type natriuretic peptide.^14^ The increasing occurrence of obesity raises the likelihood of more individuals being at risk of developing HF later on. Obesity can lead to HF by causing modifications in the cardiac system’s blood flow, shape, operation, and electrical conductivity, which encourages problems with the endothelium and blood vessels as well as metabolic imbalances and leads to insulin resistance, the release of adipokines and inflammatory markers, and cardiac lipotoxicity. Additionally, it can increase the risk of other HF risk factors, such as OSA and obesity hypoventilation syndrome. It is crucial to comprehend these mechanisms to effectively prevent HF. To develop weight-loss guidelines for HF management, it is necessary to understand the paradoxical associations between obesity and HF.^15^

Obese patients frequently experience high-output HF, which is distinguished by elevated cardiac output, reduced systemic vascular resistance, and increased oxygen consumption. This type of HF typically occurs in individuals with chronic severe anemia, hyperthyroidism, pregnancy, arterio-venous fistulae, and liver disease. Nevertheless, the precise causes of high-output HF in obese patients are not entirely clear. The complexity of addressing obesity-related HF arises from the interplay of multiple neurohormonal systems, metabolic factors, hemodynamic alterations, and various inflammatory mediators. Moreover, the absence of substantial evidence supporting the efficacy of clinical management strategies further compounds the challenge.^16^

Individuals with severe obesity experience changes in blood flow that increase the likelihood of alterations in the shape and functioning of the heart, potentially leading to HF onset. The coexistence of systemic hypertension, sleep apnea, and hypoventilation, which are frequently associated with severe obesity, may also contribute to the development of HF in affected individuals. The resulting syndrome is referred to as obesity cardiomyopathy.^17^

## The role of major adipokines in heart failure

**Figure 2:**
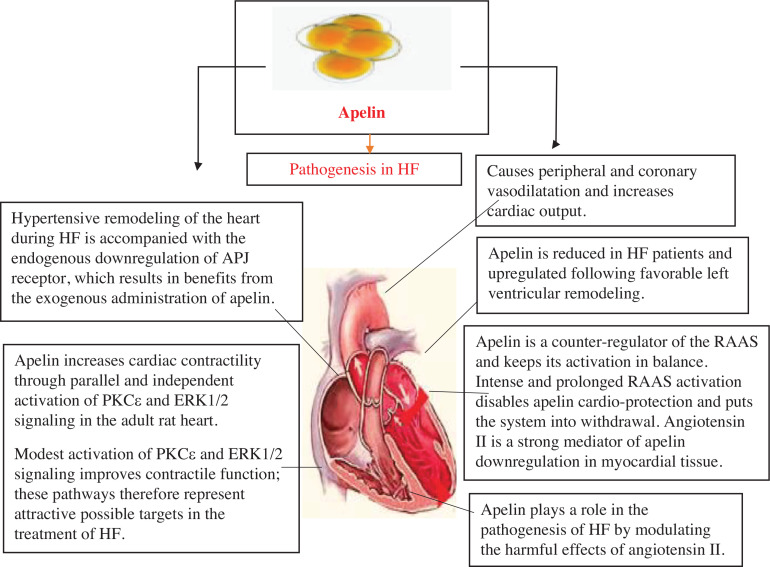
Apelin causes pathogenesis of HF. *Abbreviations:* APJ, apelin receptor; ERK1/2, extracellular signal-regulated kinase 1/2; HF, heart failure; PKCε, protein kinase Cε; RAAS, renin–angiotensin–aldosterone system.

**Figure 3:**
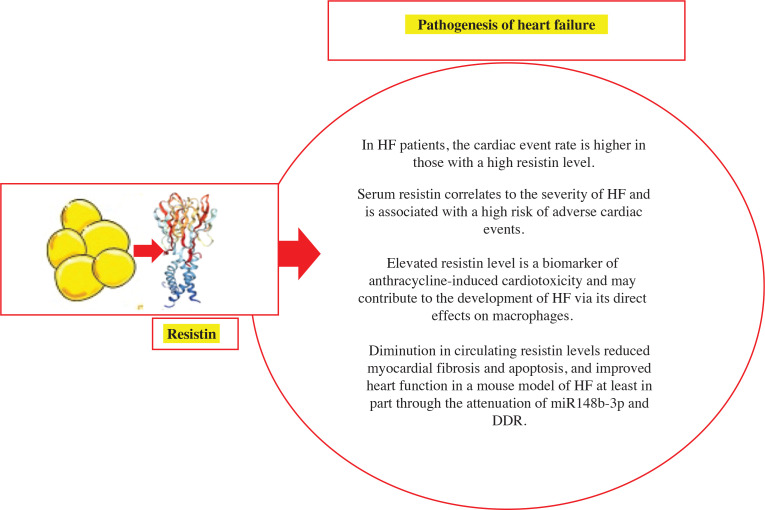
Resistin causes pathogenesis of HF. *Abbreviations:* DDR, DNA damage response; HF, heart failure.

**Figure 4:**
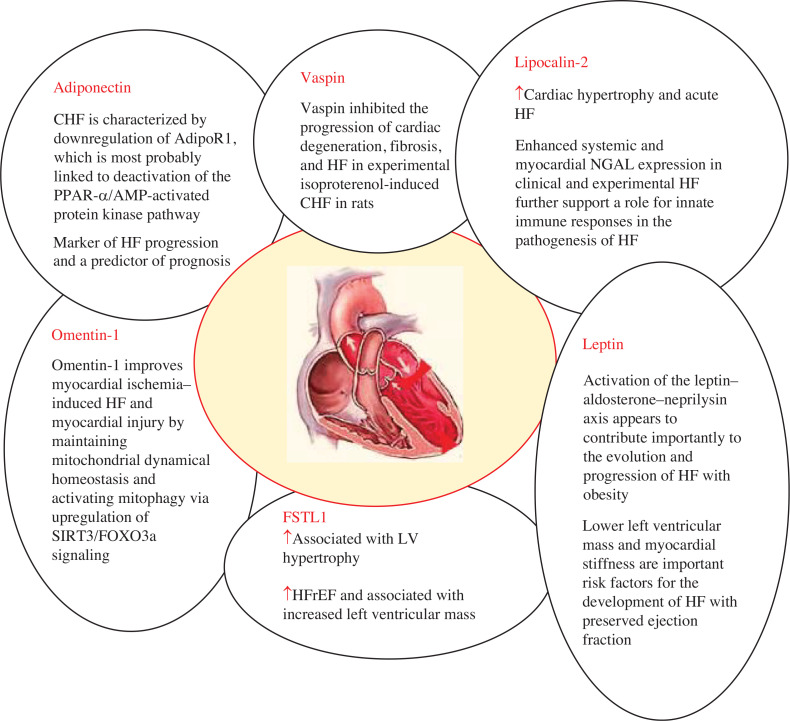
Adipokines including adiponectin, vaspin, lipocalin-2, omentin-1, leptin, and FSTL1 cause pathogenesis of heart failure. ↑ denotes increased levels. *Abbreviations:* AdipoR1, adiponectin receptor 1; CHF, chronic heart failure; FSTL1, follistatin-like 1; HF, heart failure; HFpEF, heart failure with preserved ejection fraction; HFrEF, heart failure with reduced ejection fraction; LV, left ventricular; NGAL, neutrophil gelatinase–associated lipocalin; PPAR-α, peroxisome proliferator–activated receptor-α.

**Figure 5:**
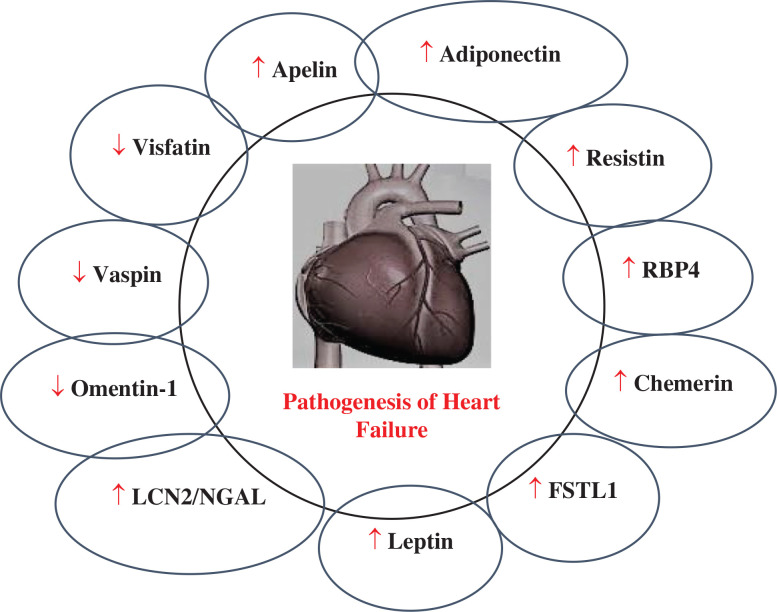
The role of circulating levels of major adipokines in HF. ↑ denotes increased levels; ↓ denotes decreased levels. *Abbreviations:* FSTL1, follistatin-like 1; HF, heart failure; LCN2, lipocalin-2; NGAL, neutrophil gelatinase–associated lipocalin; RBP4, retinol-binding protein 4.

Apelin, adiponectin, chemerin, resistin, RBP4, vaspin, visfatin, leptin, omentin-1, LCN-2, and FSTL1 all play a pathophysiological role in HF, as explained in **Figures 2–4**. The role of circulating levels of major adipokines in HF is explained in **Figure 5**.

### Apelin

The apelin receptor, also known as AR or APJ, is a type of G-protein–coupled receptor belonging to class A. It was first discovered in 1993 through homology cloning by O’Dowd et al.^18^ Initially, the receptor was considered an “orphan” until its natural ligand apelin was identified in 1998 by Tatemoto et al.^19^ When activated by the apelin peptide, its natural ligand, AR, can produce various effects on the body’s physiological functions, such as regulating the constriction and dilation of blood vessels, enhancing the heart muscle’s contractility, promoting the growth of new blood vessels, and maintaining balance in energy metabolism and fluid levels. AR is linked to various diseases like CVD, diabetes, obesity, and cancer, which makes it a potential target for treatment. Despite its significance, the exact functions of AR signaling are not yet fully comprehended. Furthermore, how peptide–AR activation occurs is not well understood. The situation is made more complicated by the fact that the AR is regulated by two naturally occurring peptide ligands, which have multiple bioactive isoforms that differ in length and distribution.^20^

Apelin is a natural molecule that binds to a newly discovered type of receptor called APJ, and it has shown significant effects on the cardiovascular system in animal studies. Giving apelin to humans in a short amount of time results in the dilation of blood vessels in the heart and throughout the body as well as an increase in the amount of blood that the heart pumps. Stimulating the APJ could be a promising new strategy for treating patients with HF.^21^

The apelin–APJ pathway is a vital molecular mechanism that helps protect the heart from injury caused by hemodynamic overload or structural damage, regardless of the cause, thereby preventing the development of HF. Apelin acts as a counter-regulator to the renin–angiotensin–aldosterone system (RAAS) and helps maintain its activation in a balanced state. However, prolonged and intense activation of the RAAS can impair apelin’s cardioprotective effects, leading to withdrawal of the system. In myocardial tissue, angiotensin II is a potent mediator of apelin downregulation. Experimental administration of apelin in HF has shown a unique combination of inotropic and vasodilatory effects with no immediate harmful effects. This forms the basis for the potential usefulness of apelin in treating HF, but further research is necessary.^22^

There is a need for new HF treatments that can safely increase the strength of heart muscle contractions and improve the amount of blood the heart pumps. Previous studies on apelin peptides have shown that activating APJ could be a promising way to enhance heart function in HF. A new, potent, and orally available APJ agonist has been discovered that closely mimics the signaling properties of (Pyr^1^) apelin-13 but is not a peptide. Giving this APJ agonist orally results in a lasting increase in cardiac output in people with heart disease, and it has effects different than those of the renin–angiotensin system inhibitor enalapril. These findings suggest that further testing of this compound, called BMS-986224, in clinical trials for HF is warranted.^23^

Apelin is a newly discovered peptide that works through the APJ receptor, which shares similarities with the angiotensin II type 1 receptor. It has peripheral vasodilatory effects, which is a potent inotrope and may influence central fluid balance. Both animal and human studies have suggested that it may play a role in the development of HF by modifying the harmful effects of angiotensin II. The levels of apelin are reduced in individuals with HF and increase after favorable LV remodeling. Apelin is present in many tissues but mostly found in the vascular endothelium. This comprehensive literature review focuses on the significant studies that have contributed to the understanding of apelin and its role in cardiovascular function and HF.^24^

When a person has HF, the expression of the APJ protein in cardiomyocytes decreases, and apelin is not upregulated to compensate. This can limit the positive inotropic effects of apelin. In hypertensive heart remodeling during HF, APJ is downregulated naturally, and exogenous administration of apelin can be beneficial. However, it has been suggested that restoring APJ expression through gene transfection could create an environment that favors the activation of the apelin–APJ signaling pathway. Further research is necessary to determine whether this would enhance inotropy and decrease peripheral resistance in a fully active state.^25^

According to research, apelin stimulates cardiac contractility by activating protein kinase Cε (PKCε) and extracellular signal-regulated kinase 1/2 (ERK1/2) signaling in the adult rat heart, which works independently and simultaneously. In addition, apelin signaling involves the activation of myosin light chain kinase downstream. The findings suggest that PKCε and ERK1/2 signaling have a role in improving contractile function, in addition to their known role in cytoprotection. Therefore, these pathways could be considered potential targets for treating HF.^26^ Apelin has been found to decrease LV preload and afterload while increasing contractile reserve, without causing hypertrophy. These findings indicate that apelin has a favorable hemodynamic profile, and therefore it may be a desirable target for pharmacological treatment in cases of HF.^27^

Apelin-13 has been shown to have beneficial effects on cardiac function in rats with HF by improving cardiac dysfunction, attenuating impaired hemodynamics, and reducing fibrosis. In addition, it has been found to reduce fibrosis in cardiac fibroblasts induced by angiotensin II through the inhibition of the phosphatidylinositol 3-kinase/protein kinase B signaling pathway, thereby attenuating oxidative stress.^28^

Another study identified alterations in the apelin/APJ system in human cardiovascular and vascular tissues affected by diseases. The reduction in receptor density observed in HF could potentially limit the beneficial inotropic effects of apelin and contribute to contractile dysfunction. The exact impact of elevated apelin levels in the atherosclerotic coronary artery on disease progression is yet to be established. These findings indicate a possible involvement of the apelin/APJ system in human cardiovascular disorders.^29^

### Adiponectin

Adiponectin was identified in 1995 as a protein produced in large quantities by 3T3-L1 adipocytes and present at high levels in mouse plasma. It has also been referred to by several other names, including ACRP30, AdipoQ, apM1, and GBP28. Interestingly, four different research groups discovered its production by white adipose tissue independently around the same time.^30–33^ Adiponectin is a protein present in adipose tissue with a specific structure consisting of 247 amino acids with a molecular weight of 30 kDa in mice or 244 amino acids with a molecular weight of 28 kDa in humans.^30,33^

Adiponectin is a hormone that regulates energy metabolism in the body, including the heart. However, its levels are found to be low in patients with diabetes, hypertension, and CVDs, making it an independent predictor of cardiovascular risk. Interestingly, recent studies have found that adiponectin has protective effects against various cardiac-related diseases that lead to HF, despite its low levels. These protective effects are attributed to its anti-inflammatory, antioxidant, and anti-apoptotic properties. Furthermore, it has been discovered that adiponectin produced locally in cardiomyocytes has functional and biologically significant effects. This locally produced adiponectin exerts its protective effects through an autocrine mechanism. The signaling pathway involved in the development of diabetic cardiomyopathy is characterized by initial myocardial energy dysregulation and lipid accumulation, which subsequently lead to contractile dysfunction. As the disease advances, cardiomyocyte damage and lipotoxicity occur in conjunction with compromised myocardial blood flow, ultimately leading to myocardial infarction and HF. Reduced levels of adiponectin in individuals with diabetes exacerbate the progression of diabetic cardiomyopathy.^34^

Adipose tissue serves not only as an energy storage site but also as an endocrine organ. One of the key adipokines secreted by adipose tissue, cardiomyocytes, and connective tissue cells in the heart is adiponectin. Adiponectin has well-known beneficial effects on metabolism and the cardiovascular system, and low levels of this protein are linked to the development of various CVDs. However, paradoxically, the levels of adiponectin gradually increase with the severity of HF, and a higher level of adiponectin is a predictor of poor prognosis. Consequently, there is increasing interest in using adiponectin as a marker of HF progression and a prognostic factor for this disease.^35^

In patients with CHF, the expression of adiponectin in skeletal muscle was significantly greater compared to recorded levels in healthy individuals, and there was also an increase in circulating adiponectin levels. The disturbances in skeletal muscle metabolism were supported by the deactivation of the peroxisome proliferator–activated receptor-α (PPAR-α)/AMP-activated protein kinase (AMPK) pathway and the downregulation of various target genes involved in fatty acid oxidation and glucose metabolism. This suggests the presence of functional adiponectin resistance as the downregulation of adiponectin receptor 1 expression and PPAR-α/AMPK deactivation occur simultaneously with the increase in adiponectin levels. It appears that the increase in adiponectin levels may be a protective mechanism to counteract adiponectin resistance and compromised energy metabolism.^36^ A single cross-sectional study demonstrated higher levels of adiponectin in patients with HF when compared to control patients.^37^

On the contrary, Frankel et al. found no correlation between adiponectin levels and HF. However, there may still be undiscovered mechanisms that contribute to the development of HF.^38^ Also, another study in a group of elderly men in Sweden did not find any link between adiponectin levels and the occurrence of HF.^39^

Recent research suggests that having a higher body mass index (BMI) may lead to better outcomes for individuals with CHF. Adiponectin, an adipocytokine that is inversely associated with BMI, is a predictor of mortality in healthy individuals. Interestingly, a high adiponectin level was also found to be a predictor of mortality in CHF patients, even after adjusting for risk markers of disease severity, likely because it is a marker of wasting. BMI was also linked to mortality, but some part of this association may be due to adiponectin and N-terminal pro-brain natriuretic peptide (NT-proBNP) levels.^40^

### Chemerin

Chemerin was first identified in 1997 using the differential display as a retinoid-responsive gene found in psoriatic skin lesions.^41^ Also known as tazarotene-induced gene 2 (*TIG2*) and retinoic acid receptor responder 2 (*RARRES2*), chemerin is a recently discovered adipokine that has been found to modulate the function of the immune system through its binding to the chemerin receptor (ChemerinR, chemokine-like receptor 1, and G-protein–coupled receptor), as reported by Roh et al.^42^ The signaling of chemerin is tightly controlled through various mechanisms, such as expression, secretion, processing, and signaling events. The proper coordination of these regulatory mechanisms is crucial for establishing the levels and location of chemerin and, ultimately, determining its activity.^43^

A recently identified adipokine called chemerin can control adipocyte development and promote the chemotaxis of dendritic cells and macrophages. Circulating chemerin is strongly linked to inflammation, obesity, metabolic syndrome, and coronary artery disease.^44,45^ Circulating chemerin has also been linked to metabolic syndrome, coronary artery disease, and inflammation in studies. Chemerin is a new biomarker in the blood that can predict major adverse cardiac events in individuals with CHF. In terms of clinical implications, serum chemerin is a new and valuable predictor of major adverse cardiac events in individuals with CHF. The presence of circulating chemerin can enhance the early identification of at-risk patients with CHF.^46^ In the same context, Menzel et al. provided the first evidence that high plasma chemerin levels are linked to a greater risk of HF.^47^

### Resistin

Resistin belongs to the hormone family called resistin-like molecules (RELMs). RELM-α and RELM-β are two other members of this family that are characterized by 10 conserved cysteine residues. Resistin and RELM-β have an additional cysteine close to their amino termini that is consistent among different species. The proteins of resistin in humans and rodents show around 60% similarity.^48–50^

Resistin was originally identified as a hormone that is specific to adipocytes, and it has been proposed that it plays a critical role in the relationship between obesity, insulin resistance, and diabetes. Even though it was first characterized in adipocytes, its expression is mostly identifiable in mononuclear leukocytes, macrophages, spleen cells, and bone marrow cells in humans. There is a growing body of evidence indicating that resistin has important regulatory functions in various biological processes such as atherosclerosis and CVD, non-alcoholic fatty liver disease, autoimmune disease, malignancy, asthma, inflammatory bowel disease, and chronic kidney disease aside from its involvement in insulin resistance and diabetes.^51^

Resistin is produced by adipose tissue in rodents, and its serum levels are increased in animal models of obesity and insulin resistance. Recent research has linked resistin to markers of inflammation and oxidative stress and has shown that it is a predictor of coronary atherosclerosis in humans. However, the significance of serum resistin has not been studied in HF patients. Takeishi et al. aimed to determine (1) whether there was a correlation between resistin and the severity of HF and (2) if resistin levels could predict clinical outcomes in HF patients. HF patients with high resistin levels had a higher rate of cardiac events than those with normal levels. Age, BMI, serum levels of resistin and brain natriuretic peptide, and loop diuretics were identified as significant predictors of future cardiac events using univariate Cox regression hazard analysis. Multivariate Cox analysis revealed that age and resistin were significant predictors of future cardiac events. It was concluded that serum resistin is associated with the severity of HF and a higher risk of adverse cardiac events in HF patients.^52^

Another study found that high levels of resistin in the bloodstream are linked to the development of HF, even after taking into account the presence of existing coronary heart disease, obesity, insulin resistance, and inflammation. This indicates that resistin may play a role in the onset of HF and presents a new pathway for understanding HF in humans.^38^

Butler et al. found that the results were consistent across subgroups that differed according to sex, race, the presence of diabetes mellitus, and the presence of both prevalent and incident coronary heart disease. In participants with information available on their LV ejection fraction (EF) at the time of HF diagnosis, resistin was found to be associated with HF risk regardless of whether the EF was reduced or preserved. Therefore, higher levels of resistin in the bloodstream were linked to an increased risk of HF in older individuals, independent of other factors.^53^

High levels of resistin are an indicator of heart damage caused by anthracycline-induced cardiotoxicity and may play a role in the development of HF by directly affecting macrophages. These findings suggest that resistin is involved in the connection between inflammation, metabolism, and heart disease.^54^

Higher levels of resistin are linked to the development of CVDs, but its specific role in HF is not fully understood. In a mouse model of HF, decreasing circulating resistin levels led to a reduction in myocardial fibrosis and apoptosis and improved heart function. This was accomplished, at least in part, by reducing the expression of miR148b-3p and DNA damage response. These findings suggest that controlling resistin levels could be a potential therapeutic strategy for treating HF induced by pressure overload.^55^

### Retinol-binding protein 4

Kanai et al. were the first to identify RBP4 in 1968 as a protein in human plasma that specifically binds to retinol and serves as its carrier in the blood. They conducted a study where they examined the plasma of individuals who were given radiolabeled retinol via injection and were able to isolate and purify the protein that had been bound to the labeled retinol, which they named RBP4.^56^ RBP4 is an approximately 21-kDa secreted protein that facilitates the movement of vitamin A (retinol) in the bloodstream. RBP4 is a well-known adipokine that helps to promote insulin resistance and obesity. Additionally, more recent clinical investigations have connected elevated RBP4 levels to several cardiovascular disorders.^57–59^

RBP4 is associated with negative consequences for the cardiovascular system. Elevated levels of circulating RBP4 have been correlated with CHF. When observing the Kaplan–Meier survival curves, it became clear that a high concentration of RBP4 can serve as a prognostic marker for major adverse cardiac events in individuals with CHF.^60^

Recent studies suggest that high levels of RBP4 in the blood may be a new risk factor for CVDs like hypertension and coronary artery disease. Evidence indicates that RBP4 can contribute to inflammation and damage to heart muscle cells, which potentially leads to HF. Majerczyk et al. investigated the correlation between plasma RBP4 levels and serum NT-proBNP, which is a strong biomarker of LV dysfunction, in an older Polish population. However, their findings suggested that RBP4 levels are influenced by the glomerular filtration rate and could not be considered an independent indicator of heart muscle dysfunction.^61^

CHF leads to an increased likelihood of developing diabetes mellitus. The decline in glycemic control and insulin resistance could be due to substances released by adipocytes. One of these substances is RBP4, which is produced by adipose tissue and has pro-diabetogenic effects. Advanced HF patients have higher levels of RBP4, but implantation of an LV assist device can reduce RBP4 levels. This suggested that RBP4 plays a role in the series of correctable metabolic abnormalities seen in advanced HF.^62^

### Vaspin

Vaspin is a newly identified adipokine derived from visceral adipose tissue (VAT). It belongs to the serpin superfamily, clade A (Serpina12), and acts as a serine protease inhibitor with the ability to increase insulin sensitivity. It is present in the VAT of Otsuka Long–Evans Tokushima fatty (OLETF) rats, which are animal models that exhibit both central obesity and type 2 diabetes.^63^ Levels of vaspin have been observed to increase significantly in both circulating blood and adipose tissue in OLETF rats at 30 weeks, which is the time at which when they exhibit the highest levels of obesity and insulin resistance. However, uncontrolled diabetes and weight loss have been found to decrease the expression of vaspin. On the contrary, the use of insulin-sensitizing drugs such as pioglitazone has been shown to restore normal expression and levels of vaspin in both serum and adipose tissue.^63,64^

It has been found that animals with insulin resistance and obesity tend to have higher levels of vaspin. Vaspin is believed to increase insulin sensitivity and have anti-inflammatory effects, potentially serving as a protective mechanism in response to decreased insulin sensitivity. In most studies involving humans, there is a correlation between markers of metabolic syndrome and the expression of the vaspin gene as well as the levels of vaspin in the blood.^65^

CHF is the outcome of most CVDs, and although treatment methods have improved over the past 20 years, morbidity and mortality rates for the condition remain high. Low levels of vaspin in the blood have been linked to an increased risk of major adverse cardiac events. However, no studies have investigated the impact of administering vaspin on HF. The vaspin might stop the progression of cardiac degeneration, fibrosis, and HF in rats with experimental isoproterenol-induced CHF.^66^

In the same way, Zhou et al. determined that low levels of vaspin are a significant predictor of hospitalization due to HF and recurrent acute myocardial infarction (AMI), even after accounting for traditional cardiovascular risk factors. The clinical significance of serum vaspin is that it serves as a significant prognostic indicator for major adverse cardiac events in patients with AMI. Additionally, measuring serum vaspin levels might improve the early identification of patients at high risk for adverse events after an AMI.^67^

### Visfatin

Visfatin is an adipokine discovered in 2004 and named for its suggested predominant production and secretion in visceral fat. It is highly conserved across animal species and has a molecular weight of 52 kDa, with its gene encoding 491 amino acids. It was later recognized as the cytokine pre–B-cell colony-enhancing factor, which was originally described in 1994 as being involved in lymphocyte maturation and inflammatory regulation. Visfatin is also known as nicotinamide phosphoribosyltransferase, the enzyme responsible for limiting nicotinamide adenine dinucleotide biosynthesis. In addition to being produced in human leukocytes and adipose tissue, visfatin is also expressed in the muscles and hepatocytes of humans and animals.^68–70^

Adipose tissue produces various secreted proteins that play crucial roles in metabolism. One such newly discovered adipocytokine is visfatin, which is highly concentrated in visceral fat, and its expression level in the blood increases during the development of obesity. Visfatin has been found to have insulin-mimetic effects and could lower plasma glucose levels by binding and activating the insulin receptor in animal models. However, studies in humans have reported conflicting results regarding its association with adiposity, insulin resistance, and dyslipidemia, which renders the role of visfatin in the development of obesity and insulin resistance unclear.^71^

The levels of visfatin and high-density lipoprotein cholesterol were found to be significantly lower in patients with HF compared to healthy individuals. The Kruskal–Wallis test revealed significant differences in the mean values of visfatin, high-sensitivity C-reactive protein, glucose, Homeostatic Model Assessment for Insulin Resistance score, and high-density lipoprotein cholesterol among the studied groups. In conclusion, the serum concentrations of visfatin were significantly lower in patients with systolic HF, especially those with more advanced New York Heart Association classes, regardless of age, anthropometric, and metabolic factors.^72^

### Leptin

Leptin, which was discovered by Friedman and his team in 1994,^73^ is the most extensively studied adipokine produced by adipose tissue. The levels of leptin increase in correlation with fat mass in individuals with obesity, which is recognized for its ability to regulate neuropeptides in the hypothalamus, thereby controlling food intake. By decreasing the activity of neurons that contain neuropeptide-Y and agouti-related protein and promoting pro-opiomelanocortin/cocaine and amphetamine-regulated transcript neurons in the arcuate nucleus of the hypothalamus, leptin acts on the central nervous system. There is a significant body of evidence suggesting that leptin also has sympatho-excitatory effects.^74^

Leptin is a 16-kDa molecule consisting of 167 amino acids. It has a tertiary structure similar to a globular protein that contains a secretory signal sequence of 21 amino acids. While white adipose tissue is the main producer of leptin, smaller quantities have been detected in various other biological tissues, including the brain, placenta, fetal tissue, stomach, muscles, bone marrow, and brown adipose tissue.^75^ When combined, the physiological effects of leptin can be harmful in conditions of HF or cardiac dysfunction. Leptin’s hemodynamic effects, including an increase in resting heart rate and blood pressure due to sympathetic nervous system activation, often lead to an increased workload on the myocardium. A lack of leptin signaling or leptin resistance increases the risk of cardiac dysfunction and HF, which is a major contributor to morbidity and mortality associated with obesity and type 2 diabetes.^76^ Activation of the leptin–aldosterone– neprilysin axis appears to play a significant role in the development and progression of HF in obese individuals, regardless of its phenotype. Targeting the harmful interactions of this specialized neurohormonal system with existing or forthcoming therapeutic medications is expected to provide unique clinical benefits.^77^

Patients with congestive HF have elevated levels of plasma leptin. The discovery of a positive association between plasma leptin and insulin concentrations implies that the increased energy expenditure observed in congestive HF patients might be caused by the insulin–leptin axis.^78^ In a moderately sized sample of older adults from the community, elevated levels of circulating leptin were associated with an increased risk of congestive HF and CVD. However, leptin did not provide additional predictive information beyond BMI. Further research is needed to fully comprehend the U-shaped correlation between leptin and mortality.^79^

Leptin levels were found to be correlated with low LV mass and myocardial stiffness, both of which are significant risk factors for the development of HFpEF. The correlation was not statistically significant in men. In women, there was an impact of leptin levels and BMI quartiles on LV mass and stiffness. Higher leptin levels were associated with reduced LV mass and stiffness in obese Black women but not in thin Black women.^80^ Similarly, there are complex relationships between leptin concentration, BMI, and LV hypertrophy/stiffness that require further investigation to determine whether leptin helps to prevent the onset of HFpEF.^81^

### Omentin-1

Omentin is a new hydrophilic adipokine consisting of 313 amino acids (35 kDa) that contains a secretory signal sequence and a fibrinogen-related domain. In its inactive form, it appears as a glycosylated trimer with a molecular weight of 120 kDa.^82,83^ Omentin-1 is the primary form present in circulation, with a concentration ranging from 100 ng/mL to 1 μg/mL in human plasma, and it has been investigated more thoroughly than omentin-2.^82^

Omentin-1 is a newly discovered secretory protein that is only expressed in VAT. It is also known as intestinal lactoferrin receptor, endothelial lectin HL-1, galactofuranose-binding lectin, or intelectin-1. A glycoprotein called mature omentin-1 has 295 amino acids and a single-linked oligosaccharide.^84,85^ Physiological concentrations of omentin-1 in humans are in the range of 100–800 ng/mL. It is well established that the expression and production of omentin-1 are affected in various pathological conditions, including obesity and insulin resistance. Furthermore, the expression of omentin-1 is changed in inflammatory conditions.^86,87^

Clinical research has shown that measuring circulating omentin-1 levels might serve as a biomarker for various conditions, such as obesity, metabolic disorders (including insulin resistance, diabetes, and metabolic syndrome), and atherosclerotic CVDs. Additionally, circulating omentin-1 levels could be used as a biomarker for bone metabolism, inflammatory diseases, cancers, sleep apnea syndrome, pre-eclampsia, and polycystic ovary syndrome. Generally, decreased levels of omentin-1 are associated with these conditions.^88^ Omentin-1 reduces myocardial ischemia–induced HF and myocardial damage by upregulating sirtuin 3/forkhead box O3a signaling, preserving mitochondrial dynamical homeostasis, and activating mitophagy. In the context of the interaction between the heart and adipose tissue, it offers support for the continued use of omentin-1 in cardiovascular disorders.^89^

Related to this, HF patients with lower serum omentin-1 levels experience worse cardiac outcomes. Measuring serum omentin-1 levels appears to be a novel prognostic indicator for risk stratification in HF patients.^90^ The correlation between omentin-1 and HF risk was linear when there was no prevalent coronary heart disease but U-shaped in patients who already had coronary heart disease. This demonstrates that the relationship between omentin-1 and the risk of HF is influenced by the presence of pre-existing coronary heart disease.^47^

### Lipocalin-2

The glycoprotein called LCN-2 is a novel adipokine that includes 198 amino acids. LCN-2 is also known as siderocalin, neutrophil gelatinase–associated lipocalin (NGAL), and uterocalin. It belongs to the lipocalin superfamily, a collection of circulating proteins that move small and hydrophobic molecules like fatty acids, steroids, retinoids, prostaglandins, and hormones.^91,92^ LCN-2 has various physiological functions, such as transporting hydrophobic ligands through cell membranes, regulating immune responses, maintaining iron levels, and promoting differentiation of epithelial cells. Despite being expressed at low levels in most human tissues, LCN-2 is abundant in more aggressive forms of cancer, such as breast, pancreatic, thyroid, ovarian, colon, and bile duct cancers. Elevated levels of LCN-2 have been linked to increased cell growth, formation of blood vessels, invasion, and spreading of cancer cells. Additionally, LCN-2 influences the degradation, allosteric changes, and enzymatic activity of matrix metalloprotease-9, which is a metalloprotease that promotes the spread and invasion of cancer cells.^93^

The likelihood of developing HF and dying from CVD is increased by cardiac hypertrophy. In some forms of cardiac hypertrophy and acute HF, levels of the neutrophil inflammatory protein known as LCN-2/NGAL were elevated. However, it is not clear what specific role LCN2 plays in the predisposition and development of hypertrophy or what genetic factors were involved.^94^ NGAL or LCN-2 is a glycoprotein known for its bacteriostatic properties. However, recent research indicates that NGAL may also play a role in cell survival, inflammation, and matrix degradation. Yndestad et al. found increased expression of NGAL in both systemic circulation and myocardium in clinical and experimental HF, suggesting that innate immune responses may contribute to the development of HF.^95^

### Follistatin-like 1

FSTL1 is a glycoprotein that belongs to the “secreted protein, acidic and rich in cysteine” family. In the scientific literature, FSTL1 is referred to by various names.^96^ FSTL1 can both protect and regenerate tissues. The level of FSTL1 in circulation increases during CVD. Healthy animal models can tolerate overexpression or lowered expression of FSTL1, but, during pathological conditions, additional FSTL1 can prevent significant cardiac damage and abnormal vascular remodeling. Conversely, a lack of FSTL1 can worsen the cardiac injury. Importantly, FSTL1 has different effects on multiple pathways depending on the type of cell and the glycosylation state of the protein. These findings suggest that FSTL1 may not only be a biomarker but also a promising candidate for the development of new therapies for CVD.^97^

FSTL1 is found in human serum outside of cells. Recent studies suggest that FSTL1 is secreted as a response to injuries caused by ischemia, and overexpression of FSTL1 can protect the heart and blood vessels. High levels of FSTL1 in the serum of patients with HF were linked to an increase in the size of the left ventricle of the heart. More research is needed to examine the role of FSTL1 as a potential biomarker in chronic systolic HF.^98^

HFpEF is a common type of HF that accounts for about 50% of all clinical presentations of HF. Its prevalence is expected to increase, but there are currently no therapies that have been proven to be effective for HFpEF. Hypertension is the leading risk factor for HFpEF, with a prevalence ranging from 60%–89% in clinical trials and human HF registries. However, treating high blood pressure alone is not enough to prevent or treat HFpEF. FSTL1, a unique member of the follistatin family of extracellular glycoproteins, was elevated in HFrEF and associated with an increase in the size of the left ventricle of the heart. These results suggest that FSTL1 may have therapeutic effects by regulating cardiac hypertrophy in HFpEF.^99^

## Conclusions

This review article concludes that adipokines like apelin, adiponectin, chemerin, resistin, RBP4, vaspin, visfatin, leptin, omentin-1, LCN-2, and FSTL1 are known to play a role in the development and progression of HF **(Figures 2–4)**. However, other adipokines, such as plasminogen activator inhibitor-1, monocyte chemotactic protein-1, nesfatin-1, and progranulin, have not yet been studied for HF. More research is needed to understand the role of adipokines like secreted frizzled-related protein 5, asprosin, FAM19a5, neuregulin 4, and others in HF. Further studies are required to find the exact mechanism of action of these adipokines in HF subjects, and therapeutic approaches are required to control the increasing prevalence of HF with obesity, which ultimately reduces the incidence of obesity-associated CVDs.
